# Bisphenol-A in Drinking Water Accelerates Mammary Cancerogenesis and Favors an Immunosuppressive Tumor Microenvironment in BALB–*neu*T Mice

**DOI:** 10.3390/ijms25116259

**Published:** 2024-06-06

**Authors:** Chiara Focaccetti, Daniela Nardozi, Monica Benvenuto, Valeria Lucarini, Valentina Angiolini, Raffaele Carrano, Manuel Scimeca, Francesca Servadei, Alessandro Mauriello, Patrizia Mancini, Zein Mersini Besharat, Michele Milella, Silvia Migliaccio, Elisabetta Ferretti, Loredana Cifaldi, Laura Masuelli, Camilla Palumbo, Roberto Bei

**Affiliations:** 1 Department of Clinical Sciences and Translational Medicine, University of Rome “Tor Vergata”, 00133 Rome, Italy; chiara.focaccetti@uniroma2.it (C.F.); monica.benvenuto@uniroma2.it (M.B.); raffaele.carrano@alumni.uniroma2.eu (R.C.); cifaldi@med.uniroma2.it (L.C.); camilla.palumbo@uniroma2.it (C.P.); 2 Department of Experimental Medicine, University of Rome “Sapienza”, 00161 Rome, Italy; daniela.nardozi@uniroma1.it (D.N.); valeria.lucarini@uniroma1.it (V.L.); valentina.angiolini@uniroma1.it (V.A.); patrizia.mancini@uniroma1.it (P.M.); zeinmersini.besharat@uniroma1.it (Z.M.B.); silvia.migliaccio@uniroma1.it (S.M.); elisabetta.ferretti@uniroma1.it (E.F.); laura.masuelli@uniroma1.it (L.M.); 3 Department of Experimental Medicine, University of Rome “Tor Vergata”, 00133 Rome, Italy; manuel.scimeca@uniroma2.it (M.S.); francescaservadei@gmail.com (F.S.); alessandro.mauriello@uniroma2.it (A.M.); 4 Department of Oncology, University of Verona, 37134 Verona, Italy; michele.milella@aovr.veneto.it

**Keywords:** endocrine-disrupting compound (EDC), breast cancer, ErbB2/*neu*-driven cancerogenesis, mouse, immunohistochemistry

## Abstract

Bisphenol-A (BPA), a synthetic compound ubiquitously present in the environment, can act as an endocrine disruptor by binding to both canonical and non-canonical estrogen receptors (ERs). Exposure to BPA has been linked to various cancers, in particular, those arising in hormone-targeted tissues such as the breast. In this study, we evaluated the effect of BPA intake through drinking water on ErbB2/*neu*-driven cancerogenesis in BALB–*neu*T mice, transgenic for a mutated ErbB2/*neu* receptor gene, which reproducibly develop carcinomas in all mammary glands. In this model, BPA accelerated mammary cancerogenesis with an increase in the number of tumors per mouse and a concurrent decrease in tumor-free and overall survival. As assessed by immunohistochemistry, BALB–*neu*T tumors were ER-negative but expressed high levels of the alternative estrogen receptor GPR30, regardless of BPA exposure. On the other hand, BPA exposure resulted in a marked upregulation of progesterone receptors in preinvasive tumors and of Ki67, CD31, and phosphorylated Akt in invasive tumors. Moreover, based on several infiltration markers of immune cells, BPA favored an immunosuppressive tumor microenvironment. Finally, in vitro cell survival studies performed on a cell line established from a BALB–*neu*T breast carcinoma confirmed that BPA’s impact on cancer progression can be particularly relevant after chronic, low-dose exposure.

## 1. Introduction

Bisphenol-A (BPA), a widely used chemical compound found in polycarbonate plastics and epoxy resins, possesses 9 out of 10 key properties to be classified as an environmental endocrine-disrupting compound (EDC) [1]. 

BPA’s ubiquitous presence in the environment, stemming from manufacturing, use, and disposal processes, leads to its release into water, soil and air, contributing to human exposure through multiple routes [2,3,4]. High temperatures or pH changes can trigger the cleavage of the ester bond that links BPA monomers and enable their migration into food and liquids [5,6,7]. BPA, in fact, leaches from products like food containers, cans, and dental materials, increasing dietary exposure. Inhalation and dermal contact further contribute to human exposure, highlighting the complex and multi-faceted aspects of BPA exposure pathways [8,9,10]. 

Although hundreds of epidemiology studies have shown the direct correlation between BPA exposure and adverse effects (obesity, diabetes, infertility, thyroid dysfunction, autism spectrum disorders, cancer, and toxicological effects in fetuses, neonates, and children), regulatory agencies worldwide do not present common guidelines and, above all, not all of them have updated guidelines for the “safe” use of this compound [1,11]. In 2010, the World Health Organization (WHO) defined the upper limit of daily mean exposure for food consumption, inhalation, or indirect ingestion of BPA for the general population [12]. Lately, supported by recent studies, the European Food Safety Authority (EFSA) suggested lowering the tolerable daily BPA dosage from 4 µg/kg body weight/day established in 2015 to 0.04 ng/kg body weight/day, but the most recent EFSA approval available by the end of 2022, fixed the tolerable daily intake at 0.2 ng/kg body weight/day [13,14]. 

Since the structure of BPA resembles that of the synthetic estrogen, diethylstilbestrol, BPA has the ability to mimic estrogens and bind to canonical and non-canonical estrogen receptors (ER) [5,15]. In fact, it not only interacts with nuclear ERsbut also activates membrane ERs and GPR30, i.e., the non-classical membrane G protein-related receptors, even at low concentrations [16]. 

Although the known primary effect of BPA relies on its estrogenic activity, it also possesses additional endocrine-disruptive functions. BPA, in fact, disrupts thyroid hormone receptor function and exhibits a moderate anti-androgenic effect by inhibiting androgen binding to the androgen receptor. Additionally, BPA can bind as an agonist to the glucocorticoid receptor and peroxisome proliferator-activated gamma receptor (PPARγ) [17].

Actually, BPA exposure has been linked to various cancers, in particular those arising in hormone-target tissues such as breast, prostate, testis, ovary, and endometrium [18,19,20]. It has been demonstrated that in males, BPA exposure at the early stage of life might abnormally regulate the proliferation, growth, and migration of cells, thus, inducing a predisposition to developing testicular and prostate cancer [21,22,23,24]. BPA also triggers the proliferation and migration of different cell types, including lung, colorectal, and liver cells [25,26]. Perinatal exposure to BPA could induce benign or malignant changes in the female reproductive system, such as cysts, which may lead to the development of uterine or ovarian tumors [27,28,29,30,31,32,33]. 

Breast cancer is the most common cancer among women [34]. Although it is primarily classified on the basis of ER, progesterone receptor (PR), and human epidermal growth factor 2 (ErbB2/*neu* or HER2) receptor expression, breast cancer is a heterogeneous disease with multiple molecular subtypes that differ in terms of both clinical behavior and response to therapy [35,36,37]. 

In this context, the different subtypes of ErbB2/*neu*-positive breast cancers are very aggressive tumors with a high risk of recurrence and poor clinical outcome. Indeed, sustained ErbB2/*neu* signaling stimulates proliferation, inhibits apoptosis, confers chemotherapy resistance, and promotes invasion and metastasis of breast cancer cells [38,39,40].

Since mammary glands are particularly sensitive to hormones, they are at greater risk of malignant transformation following exposure to environmental EDCs such as BPA [18,41]. Indeed, this compound can induce breast carcinogenesis through both estrogen-dependent and -independent pathways, epigenetic changes, and DNA damage [42]. Several studies highlighted that breast cancer cells exposed to BPA show increased proliferation and migration, also through the activation of the GPR30/EGFR/ERK signaling pathway [43,44]. Moreover, BPA exposure can lead to the activation of other signaling pathways, such as Akt/PI3K or JAK-STAT pathways, and to the abnormal regulation of p53 expression in breast cancer [5,45]. Furthermore, besides its effects on cancer cells, BPA has been reported to affect the properties of the tumor microenvironment and to alter immune surveillance, thereby potentially exerting additional detrimental effects on breast cancer progression [46,47,48].

Given these premises, the aim of this study was to evaluate the effect of BPA intake through drinking water on ErbB2/*neu-*driven cancerogenesis in BALB–*neu*T mice. These mice are transgenic for a mutated version of the rat ErbB2/*neu* receptor gene and develop multifocal carcinomas in all mammary glands with 100% penetrance [49,50,51]. In this breast cancer model, the effect of the compound on the time of tumor onset, the number of tumors per mouse, and mice survival was investigated, as well as its impact on the expression of cell proliferation, angiogenesis, apoptosis, and immune cell infiltration markers in tumor tissues. Our findings demonstrate that BPA in drinking water accelerates ErbB2/*neu*-mediated mammary cancerogenesis in the transgenic BALB–*neu*T mouse model and favors an immunosuppressive tumor microenvironment.

## 2. Results

### 2.1. Effect of BPA on ErbB2/neu-Mediated Mammary Carcinogenesis in the BALB–neuT Model

**Figure 1:**
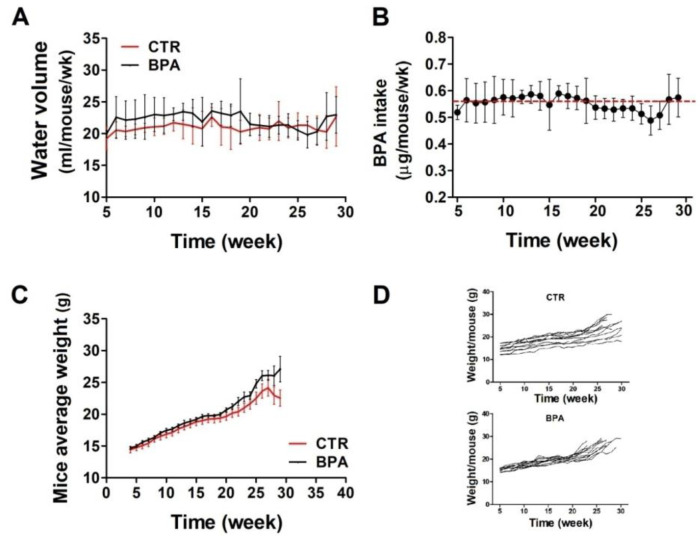
BPA intake and growth monitoring in BALB–*neu*T mice. (**A**) Volume of water drunk by BPA-treated mice compared with CTR-treated mice. (**B**) Average BPA intake per week. The red dashed line indicates the average amount of BPA ingested over the whole observation period. (**C**,**D**) The difference in weight between BPA- and CTR-treated mice, shown as cumulative (**C**) or per single mouse (**D**).

To evaluate the effect of BPA on ErbB2/*neu*-mediated mammary carcinogenesis, the BALB–*neu*T mouse model, transgenic for an activated rat *neu* oncogene under the control of the mouse mammary tumor virus promoter, was employed. Beginning at weaning (3 weeks of age), BPA (25 µg/L) or its vehicle (0.08% EtOH) was supplied in the drinking water of BALB–*neu*T female mice ad libitum. At weekly intervals, the volume of water drunk in each cage was calculated as average. There were no significant differences in the amount of water drank by the two groups of mice: those in the control group (CTR) drank an average of 21.1 ± 0.8 mL of EtOH-containing water weekly, while those drinking BPA-containing water ingested an average of 22 ± 1.2 mL of water per week (Figure 1A). Accordingly, mice supplied with BPA-containing water ingested an average of 0.56 µg/mouse/week of BPA, equivalent to 4 µg/kg body weight/day (Figure 1B). 

At weekly intervals, mice were weighed to reveal whether the compound affected their growth. Mouse weight, monitored up to around 30 weeks, was similar in both groups, thus suggesting that BPA did not induce evident health effects (Figure 1C,D).

**Figure 2:**
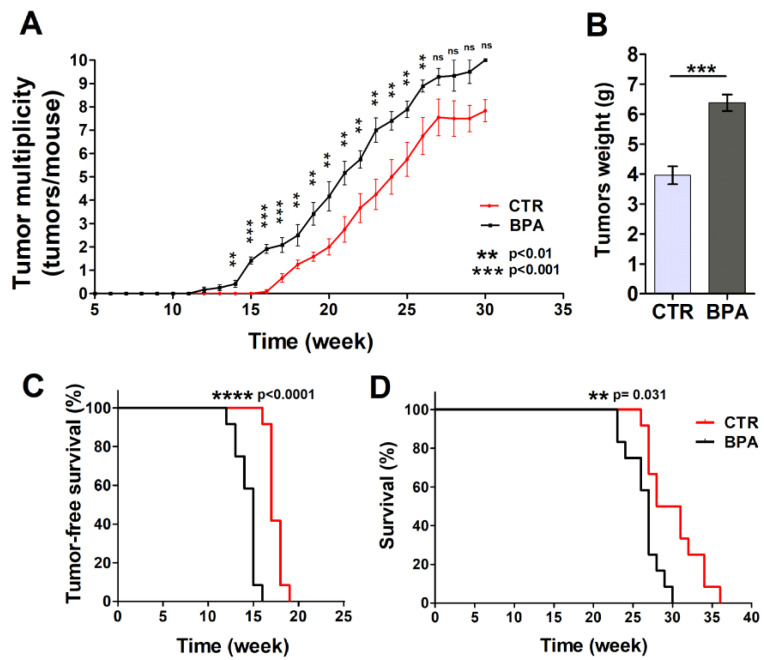
Effect of BPA on tumor development and mice survival. (**A**) Average tumor multiplicity in mice drinking BPA- or vehicle-containing water from weaning (3 weeks of age). Average tumor multiplicity was defined by the cumulative number of tumors/number of animals, using an upper limit of 10 tumors/mouse. (**B**) Weight of tumors collected from age-matched BPA-treated or CTR-treated mice (n = 5). Results are expressed as mean ± SD values (*** *p* ≤ 0.001 vs. CTR). (**C**) Tumor-free survival and (**D**) overall survival of mice drinking BPA- or vehicle-containing water from weaning.

Mammary glands were analyzed each week to detect the appearance of tumors. Tumors were palpable at week 12 in BPA-treated mice (9 weeks of BPA ingestion), while the first appearance of tumors was recorded at 16 weeks of age in the CTR group (Figure 2A,B). The difference in the number of tumors per mouse between BPA- and CTR-treated mice became significant at 14 weeks of age (*p* < 0.01) when the presence of tumors was still not evident in CTR mice and remained significant until week 26. In particular, mice drinking BPA developed twice as many tumors as CTR mice up to 21 weeks of age (20th week: BPA 4.2 tumors per mouse vs. CTR 2 tumors per mouse, *p* < 0.01; 21st week: BPA 5.2 tumors per mouse vs. CTR 2.75 tumors per mouse; *p* < 0.01) (Figure 2A). Later, the number of tumors per mouse remained higher in BPA- vs. vehicle-treated mice up to 26 weeks of age (22nd week: BPA 5.75 tumors per mouse vs. CTR 3.71 tumors per mouse, *p* < 0.01; 23rd week: BPA 7.0 tumors per mouse vs. CTR 4.25 tumors per mouse, *p* < 0.01; 24th week: BPA 7.4 tumors per mouse vs. CTR 5.0 tumors per mouse, *p* < 0.01; 25th week: BPA 7.9 tumors per mouse vs. CTR 5.75 tumors per mouse, *p* < 0.01; 26th week: BPA 8.9 tumors per mouse vs. CTR 6.75 tumors per mouse, *p* < 0.01). The weight of tumors collected from age-matched mice was also recorded, highlighting an increased weight of the tumors from BPA-treated mice as compared to those from CTR-treated mice (Figure 2B). 

Tumor-free survival was 17 weeks in the CTR group, while it decreased to 15 weeks in BPA-treated mice (*p* < 0.0001). All animals receiving BPA developed at least one mammary tumor at 16 weeks of age, whereas all CTR animals developed at least one tumor at 19 weeks of age (Figure 2A,C). In parallel, BPA- and CTR-treated mice significantly diverged in their survival (*p* = 0.0031) (Figure 2D). Indeed, CTR-treated mice showed an average survival of 29.5 weeks, while BPA-treated mice showed an average survival of 27 weeks. All BPA-treated mice were sacrificed between 23 and 30 weeks due to the presence of tumors in all mammary glands (Figure 2D). 

Based on these findings, BPA intake accelerated ErbB2/*neu* mammary cancerogenesis in the BALB–*neu*T mouse model, resulting in an increased number of tumors per mouse, an increased tumor weight, and a concurrent decrease in tumor-free and overall survival. 

### 2.2. Histological Analysis and Receptor Status of Mammary Tumor Tissues from BPA-Treated Mice

**Figure 3:**
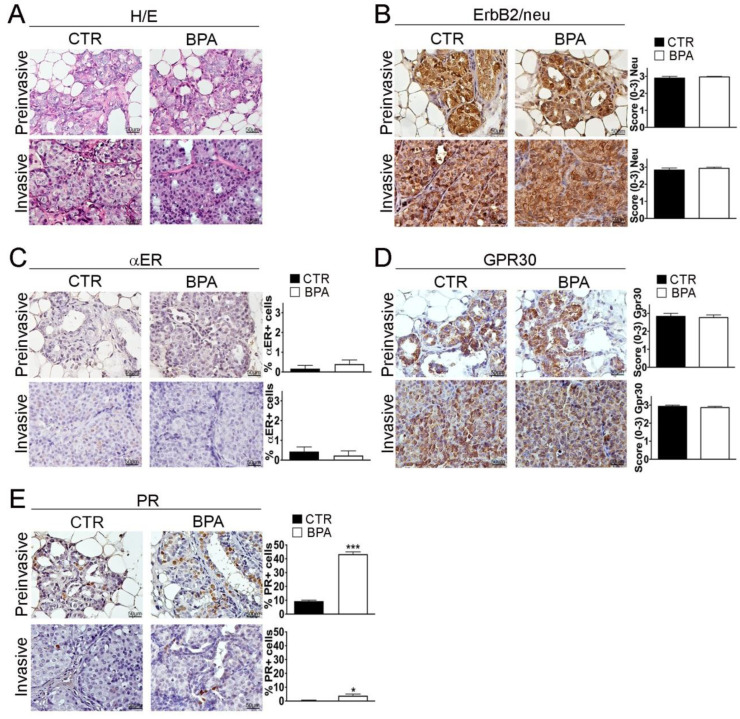
Histological analysis and receptor status of mammary tumor tissues from BALB–*neu*T mice. Mammary tissues were collected from BPA-treated and untreated (CTR) BALB–*neu*T mice at the preinvasive or invasive stage of tumor progression (n = 3). (**A**) Hematoxylin/eosin (H/E) staining. (**B**–**E**) Immunostaining for (**B**) ErbB2/*neu*, (**C**) αER, (**D**) GPR30, (**E**) PR, scored as described in Section 4. Tissue sections were counterstained with hematoxylin. The results are expressed as the mean  ±  SD values of three independent experiments performed in triplicate (* *p* ≤ 0.05; *** *p* ≤ 0.001 vs. CTR). Images were acquired with an OLYMPUS BX53 microscope (Hachioji, Tokyo, Japan) (original magnification 200×). Lower magnification images are available in Supplementary Figure S1.

BALB–*neu*T female mice develop multifocal mammary lesions that reproducibly progress from non-invasive to invasive breast carcinomas [49,50]. As previously described by Di Carlo et al., in the early stage of life, the mammary tissue of BALB–*neu*T female mice is characterized by atypical hyperplasia of small lobular ducts. Starting from the 11th week, the tissue presents features typical of lobular carcinoma “in situ” such as an occlusive intralobular growth of epithelial cells. Afterwards, although there was no evidence of a typical linear arrangement of neoplastic cells around lobules and normal ducts, morphological alterations typical of alveolar and solid variants were detected [52,53]. Notably, atypical lobular hyperplasia and lobular carcinoma “in situ” are part of a spectrum of preinvasive lesions of lobular breast cancer, distinguished from each other solely by the quantitative extension of the terminal duct lobular units (TDLUs) involved (less than half of the acini in TDLUs for the atypical lobular hyperplasia, more than half of the acini for the lobular carcinoma “in situ”) [54], therefore, in this study, the two categories were grouped into a single entity called “preinvasive” lobular neoplasia. Accordingly, hematoxylin/eosin staining and immunohistochemistry (IHC) were performed on sections from mammary tumor tissues collected at the preinvasive (11 weeks) or invasive stage (30 weeks) of tumor progression from both BPA- and CTR-treated mice. No major histopathological differences were found between CTR- and BPA-treated mice at the different stages investigated (Figure 3A and Supplementary Figure S1). CTR- and BPA-treated mice showed uniformly high levels of ErB2/*neu* expression in all lesions, regardless of tumor stage (Figure 3B). 

Hormone receptor expression was then analyzed. Overall, the percentage of cells positive for the BPA-target receptor αER was lower than 1% at both tumor stages, with no significant differences between CTR- and BPA-treated mice (Figure 3C). In both groups of mice, the non-classical estrogen receptor and BPA-target GPR30 were instead expressed at high levels in preinvasive as well as in invasive lesions (Figure 3D). As for PR, in CTR mice, the percentage of cells positive for its expression showed a reduction from about 10% at the preinvasive stage to less than 1% at the invasive stage. Interestingly, in BPA-treated mice, PR expression was also downregulated with tumor progression, but the percentage of PR-positive cells was significantly higher as compared to that of CTR-treated mice at both the preinvasive (*p* < 0.001) and invasive stage (*p* < 0.05) (Figure 3E). 

### 2.3. Expression of Tumor Progression Markers in Mammary Tumor Tissues from BPA-Treated Mice

**Figure 4:**
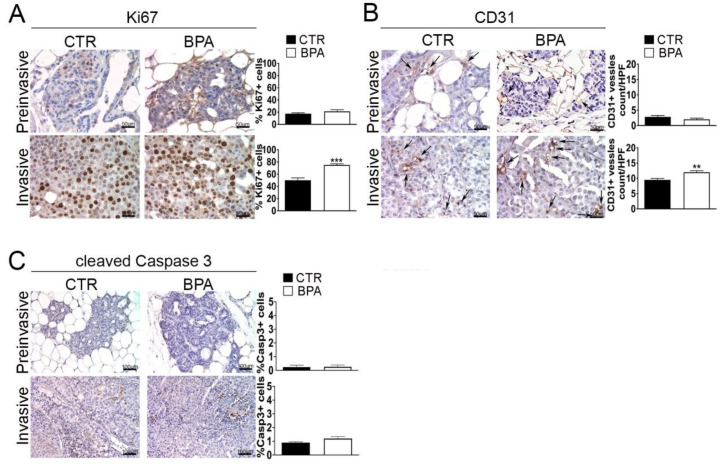
Expression of proliferation, neoangiogenesis and apoptosis markers in mammary tumor tissues from BALB–*neu*T mice. Mammary tissues were collected from BPA-treated and untreated (CTR) mice at the preinvasive or invasive stage of tumor progression (n = 3) and immunostained for markers of (**A**) proliferation (Ki67), (**B**) neoangiogenesis (CD31), and (**C**) apoptosis (cleaved caspase 3). The immunostaining was scored as described in Section 4. Tissue sections were counterstained with hematoxylin. The results are expressed as the mean ± SD values of three independent experiments performed in triplicate (** *p* ≤ 0.01; *** *p* ≤ 0.001 vs. CTR). Arrows indicate CD31-positive vessels. Images were acquired with an OLYMPUS BX53 microscope (original magnification 200×).

The expression of Ki67 and CD31, markers of proliferation and neoangiogenesis, respectively, was investigated by IHC in tumor tissues from BPA- and CTR-treated mice. The percentage of cells expressing the Ki67 proliferation marker was similar (~20%) in preinvasive lesions from BPA- and CTR-treated mice. On the other hand, the percentage of Ki67-positive cells was increased to a higher level (*p* < 0.001) in invasive lesions from BPA-treated (~70%) as compared to CTR-treated mice (~50%) (Figure 4A). A similar trend was observed for the expression of CD31: the number of CD31-positive vessels, which was comparably low in preinvasive lesions from the two groups of mice, increased to a greater extent in invasive lesions from BPA-treated mice vs. those from CTR-mice (*p* < 0.01) (Figure 4B).

A small number of apoptotic cells, identified by positive immunostaining for the large fragment of activated caspase 3, was revealed in invasive tumors from both BPA- and CTR-treated mice without significant differences between the two groups (Figure 4C). 

**Figure 5:**
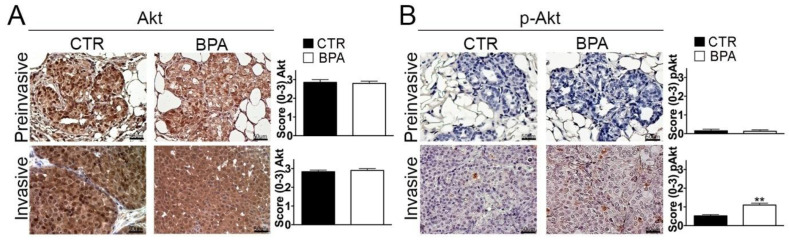
Expression of total and phosphorylated Akt in mammary tumor tissues from BALB–*neu*T mice. Akt (**A**) and phospho-Akt (p-Akt) (**B**) immunostaining of mammary tumor tissues collected from BPA-treated and untreated (CTR) mice at the preinvasive or invasive tumor stage (n = 3). The immunostaining was scored as described in Section 4. Tissue sections were counterstained with hematoxylin. The results are expressed as the mean  ±  SD values of three independent experiments performed in triplicate (** *p* ≤ 0.01 vs. CTR). Images were acquired with an OLYMPUS BX53 microscope (original magnification 200×).

In light of its pivotal role in promoting breast cancer cell survival and proliferation [55], the expression of Akt and its phospho-activated form (p-Akt) was also investigated. While Akt was similarly expressed in preinvasive and invasive lesions from both BPA- and CTR-treated mice (Figure 5A), a significant increase of p-Akt levels was observed in invasive lesions from BPA-treated mice (*p* < 0.01) (Figure 5B). 

### 2.4. Evaluation of Tumor Immune Microenvironment in BPA-Treated Mice

**Figure 6:**
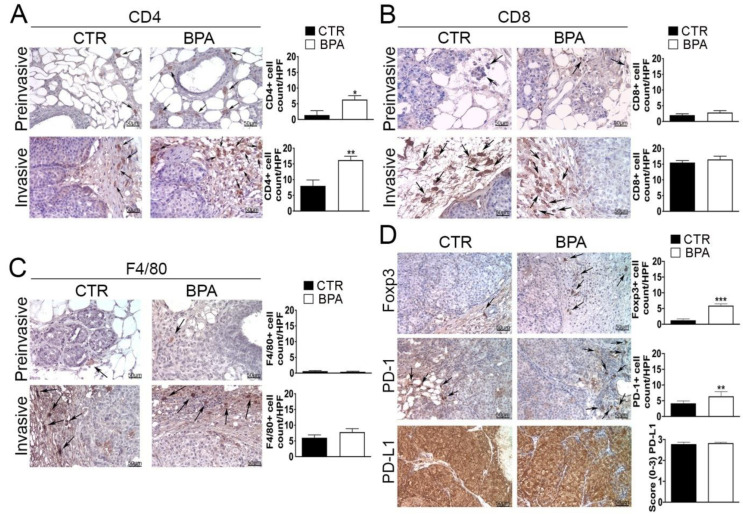
Expression of immune cell markers on mammary tumor tissues from BALB–*neu*T mice. Mammary tissues were collected from BPA-treated and untreated (CTR) mice at the preinvasive or invasive stage of tumor progression (n = 3) and immunostained for (**A**) CD4, (**B**) CD8, and (**C**) F4/80. (**D**) Tissues collected from BPA-treated and untreated (CTR) mice at the invasive stage were immunostained for Foxp3, PD-1, and PD-L1. The immunostaining was scored as described in Section 4. Tissue sections were counterstained with hematoxylin. The results are expressed as the mean  ±  SD values of three independent experiments performed in triplicate (* *p* ≤ 0.05; ** *p* ≤ 0.01, *** *p* ≤ 0.001 vs. CTR). Arrows indicate positive CD4 (**A**), CD8 (**B**), F4/80 (**C**), and Foxp3 or PD-1 (**D**) cells. Images were acquired with an OLYMPUS BX53 microscope (original magnification 200×).

Next, immunohistochemical studies were performed in order to investigate the impact of BPA intake on the tumor immune microenvironment. The analysis of the immune cell infiltrate showed that both preinvasive and invasive lesions from BPA-treated mice had an increased number of CD4^+^ T lymphocytes as compared to those of CTR-treated mice (*p* < 0.05 and *p* < 0.01, respectively) (Figure 6A). Conversely, no significant differences in the number of cells positive for CD8 or for the macrophage marker F4/80 were observed between the two groups of mice at either stage of the lesions (Figure 6B,C). 

Tissue samples from tumors at the invasive stage were also used to assess the expression of markers of an immunosuppressive microenvironment, including the regulatory T cells (Tregs) marker Foxp3, the immune inhibitory receptor programmed cell death-1 (PD-1) and its ligand PD-L1 [56,57,58,59]. In fact, invasive lesions from BPA-treated mice showed an increased number of Foxp3 and PD-1 positive cells (*p* < 0.001 and *p* < 0.01, respectively), whereas PD-L1 was constitutively expressed at high levels and in a high percentage of cells in tumors from both groups of mice (Figure 6D). 

**Figure 7:**
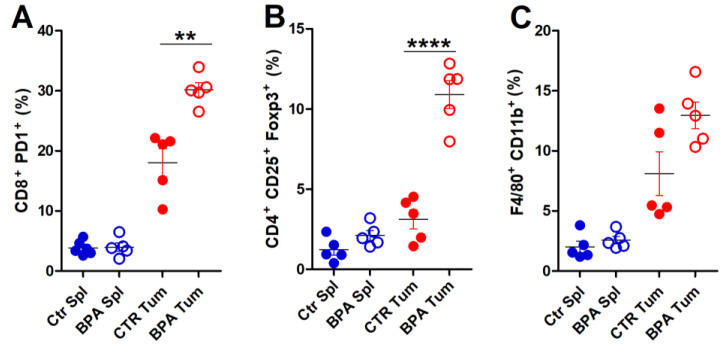
Flow cytometric analysis of immune cells extracted from invasive tumors of BALB–*neu*T mice. Cells were extracted from spleens (Spl) and invasive tumors (Tum) of BPA- and CTR-treated mice (n = 5) and processed for flow cytometric analysis. (**A**) Exhausted T lymphocytes (CD8^+^PD-1^+^), (**B**) Treg cells (CD4^+^CD25^+^Foxp3^+^), and (**C**) macrophages (F4/80^+^CD11b^+^) were evaluated. The results are expressed as mean  ±  SD values (** *p*  ≤  0.01; **** *p*  ≤  0.0001).

To provide a deeper characterization of the immune infiltrate populating the tumor microenvironment, flow cytometric analysis of immune cells extracted from tumor tissues was performed on invasive lesions of BPA- and CTR-treated mice (Figure 7). A significant increase in the frequency of exhausted, CD8^+^PD-1^+^ T lymphocytes (Figure 7A) and of CD4^+^CD25^+^Foxp3^+^ Treg cells (Figure 7B) was found in tumors from BPA-treated as compared to CTR-treated mice. The frequency of F4/80^+^CD11b^+^ macrophages [60] also showed an increasing trend in tumors from BPA-treated mice (Figure 7C). By comparison, CD8^+^PD-1^+^ T lymphocytes, CD4^+^CD25^+^Foxp3^+^ Treg cells, and F4/80^+^CD11b^+^ macrophages collected from spleens of BPA- and CTR-treated mice showed similarly low frequencies (Figure 7A,C).

### 2.5. Dose-Dependent Effects of BPA on Survival of ErbB2/neu-Driven Breast Cancer Cells In Vitro

**Figure 8:**
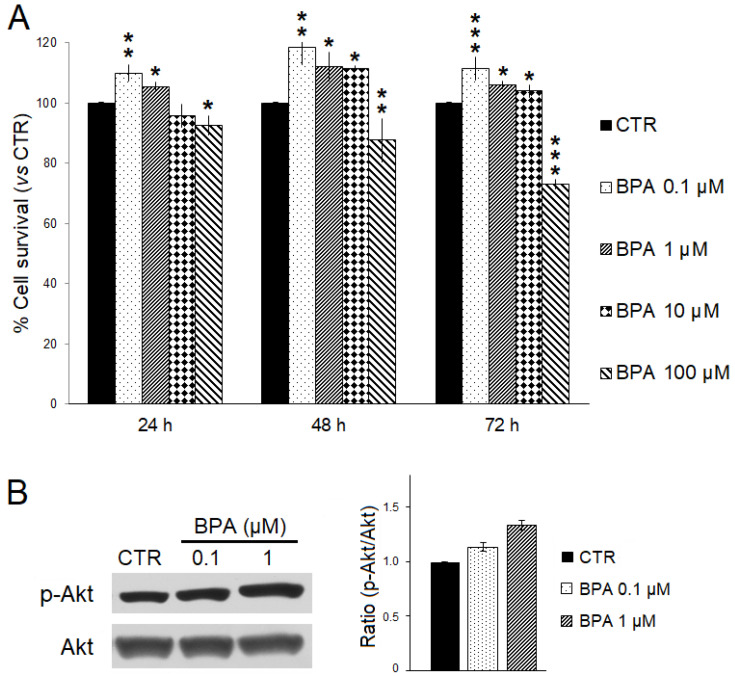
Effects of BPA on TUBO breast cancer cells in vitro. (**A**) Cell survival was evaluated by SRB assay after 24, 48, and 72 h of treatment with BPA (0.1, 1, 10, and 100 μM) or EtOH 0.5% as the vehicle (CTR). The percentage survival of BPA-treated TUBO cells was calculated relative to that of EtOH-treated control cells. Results are expressed as the mean ± SD of three independent experiments performed in triplicate. Statistical significance was calculated with one-way ANOVA (* *p* ≤ 0.05, ** *p* ≤ 0.01, *** *p* ≤ 0.001). (**B**) Effect of BPA on Akt expression and activation. Western blotting analysis was performed on TUBO cells treated with BPA (0.1–1 µM) or EtOH 0.5% (CTR) for 24 h. The levels of phospho-Akt (p-Akt) were compared with those of total Akt. Densitometric ratios and statistical analysis are reported. Data are expressed as the mean ± SD of two independent experiments.

The effects of BPA on ErbB2/*neu*-driven breast cancer cell survival were investigated in vitro using TUBO cells, a cell line previously established from a BALB–*neu*T mouse breast carcinoma, and SRB assay (Figure 8A) [49,61]. TUBO cells were incubated with increasing BPA concentrations (range: 0.1–100 μM) or EtOH as the control for 24, 48, and 72 h. As compared to control, a significant dose- and time-dependent reduction in cell survival was observed when TUBO cells were treated with 100 µM BPA. Conversely, lower concentrations of BPA (0.1–10 µM) significantly increased TUBO cancer cell survival. Moreover, the lowest BPA concentration appeared to be the most effective in increasing cancer cell survival. After 72 h of treatment, the survival of TUBO cells treated with BPA 0.1 µM was significantly higher than that observed with BPA 1 µM (110% vs. 105%, *p* < 0.05) or BPA 10 µM (110% vs. 104%, *p* < 0.001). Reasonably, the small survival differences observed after short-term BPA exposure would be amplified after chronic, long-term exposure. 

We also evaluated by Western blotting analysis whether BPA treatment could affect expression and phosphorylation levels of the pro-survival kinase Akt. Consistent with the findings obtained in BALB–*neu*T mice studies, BPA increased the phospho-activation of Akt in TUBO cells (Figure 8B). 

## 3. Discussion

The results presented herein first demonstrate that chronic exposure to a low dose of BPA through drinking water [18], corresponding to an intake of 4 µg/kg body weight/day, accelerates ErbB2/*neu* mammary cancerogenesis in BALB–*neu*T female mice. In particular, BPA-treated mice showed a decreased tumor-free survival, developing at least one mammary tumor 3 weeks earlier than vehicle-treated mice, and a reduced overall survival. 

Previous studies performed in different rodent models have shown that in utero or postnatally, BPA exposure can increase susceptibility to chemically-induced mammary carcinogenesis and that, in some cases, BPA can increase mammary tumor incidence even when administered alone [18]. The impact of chronic BPA intake was also investigated in a study performed using transgenic mice that spontaneously developed mammary tumors driven by the overexpression of wild-type ErbB2/*neu* (MMTV-ErbB2 mice) but after a longer latency and with a lower multiplicity as compared to BALB–*neu*T mice [62]. In this study, similar to our results, BPA accelerated mammary cancerogenesis. Moreover, BPA-treated MMTV-ErbB2 mice also showed an increased incidence of lung metastases [63]. 

In addition to the findings on mice survival, we show here that BALB–*neu*T mice developed tumors that were ER-negative at both the preinvasive and invasive stages regardless of BPA exposure. In this respect, guidelines recommend positive staining in ≥1% of tumor cells as the threshold for defining an αER-positive status [64], whereas the tumors from CTR and BPA-treated mice had a percentage of αER-positive cells lower than 1% at both the investigated stages. On the other hand, the alternative estrogen receptor GPR30 was highly expressed at the preinvasive and invasive tumor stages in tissues from both mice groups. Therefore, even though in BALB–*neu*T mice, αER expression appears to be lost at an early stage of ErbB2/*neu*-driven carcinogenesis, BPA can nonetheless promote tumor progression by acting via GPR30. Indeed, besides the well-known role of αER-mediated genomic and non-genomic pathways in enhancing breast cancer cell proliferation, survival, and invasion [65,66], a key role in estrogen-dependent responses and in the progression of breast cancer is played by GPR30 [67,68,69,70], and it has been previously demonstrated that GPR30 can mediate BPA estrogenic signals in ER-negative breast cancer cells [44,71]. While it is reported that about 60% of human breast cancers are positive for GPR30 expression [68], that mammary tumors of BALB–*neu*T mice express high levels of this alternative ER has not been reported before to our knowledge and highlights that these transgenic mice represent a valuable model to investigate the crosstalk between GPR30 and ErbB2/*neu* in vivo [72]. 

At variance with αER and GPR30, PR levels showed significant changes related to both tumor stage and BPA-exposure. In fact, BPA exposure caused a marked upregulation of PRs in preinvasive tumors and a modest but significant PR upregulation in invasive tumors. In particular, in preinvasive tumors from CTR mice, PRs were expressed in about 10% of cells, while in those from BPA-treated mice, more than 40% of cells were PR-positive. Moreover, in invasive tumors from both mice groups, PRs were strongly downregulated, but the percentage of PR-positive cells remained higher in tissues from BPA-treated than in those from CTR mice. 

That BPA could induce an increase of PR expression in mouse mammary tissues has been previously reported, but given that PR expression is induced by ER activity, it has been mainly regarded as a downstream result of ER’s activation by the endocrine disruptor [73,74]. However, when considering that the mammary tumors of BALB–*neu*T mice are ER-negative at both the preinvasive and invasive stages, the mechanisms responsible for the upregulation of PR by BPA at these stages of tumor progression will deserve further investigations. Indeed, the increase of PR-positive cells could be a long-term manifestation of the effects induced by BPA at an earlier stage of tumor development, preceding the loss of ER expression by tumor cells [27]. Still, it is also possible that different ER-independent mechanisms could mediate the effects of BPA on PR expression [75,76,77].

Although the role of PR in breast cancer has been debated, with some reports indicating that the activation of this receptor could restrain tumor cell proliferation [77,78,79], a growing body of evidence supports its role in promoting breast carcinogenesis [80,81,82]. For instance, by engrafting human breast cancer cells with either PR downmodulation or ectopic expression into the milk ducts of immunodeficient mice, it has recently been demonstrated that PR is required for cancer growth and that its activation is sufficient to drive proliferation as well as invasion and metastasis [83].

The expression levels of Ki67 and CD31, markers of proliferation and neoangiogenesis, respectively, were also increased in invasive tumors of BPA-treated BALB–*neu*T mice, along with that of p-Akt. Notably, Akt signaling, which has a well-established role in promoting breast cancer cell survival and proliferation [55], may also be involved in the post-transcriptional downregulation of PR levels observed at the invasive stage [77].

The findings reported herein further demonstrate that chronic exposure to BPA at low doses can participate in ErbB2/*neu*-driven mammary tumor progression by acting on the tumor immune microenvironment. In this regard, we first investigated the frequency of CD4^+^, CD8^+^, and F4/80^+^ cells in both preinvasive and invasive tumors from BALB–*neu*T mice and observed that the number of cells positive for CD8 or for the macrophage marker F4/80 were similar in CTR and BPA-treated mice at either stage of the lesions, whereas the lesions from BPA-treated mice had an increased number of CD4^+^ cells. In particular, the number of CD4^+^ cells infiltrating the tumors of BPA-treated mice was about three times that found in tumors of CTR mice at the preinvasive stage and about two times that of CTR mice at the invasive stage. This finding may already indicate an adverse effect caused by BPA exposure since, according to some studies, infiltration of breast cancer tissues by CD4^+^ T lymphocytes is associated with a more aggressive tumor phenotype and lymph node metastasis and has a negative impact on patients’ prognosis [84,85,86]. However, there is no general consensus on this matter [87,88]. On the other hand, more compelling evidence that BPA can favor an immunosuppressive tumor microenvironment in the BALB–*neu*T model is provided by the marked increase of Foxp3^+^ cells observed in invasive tumors of BPA-treated mice. In fact, Foxp3 is regarded as the most specific marker for regulatory Tregs, i.e., a T cell subset that plays a crucial role in maintaining immune homeostasis in physiological conditions but is also able to promote tumor immune evasion by suppressing anti-tumor lymphocyte functions [56,89,90,91]. Specific flow cytometric analysis for CD4^+^CD25^+^Foxp3^+^ cells highlighted the increased frequency of Treg cells in tumors extracted from BPA-treated mice. Studies performed in different cohorts of breast cancer patients have shown that the presence of Foxp3^+^ cells is increased in high-grade tumors and is associated with increased risk of relapse and decreased survival [86,92,93]. Interestingly, similar to our results, in a different mouse model of breast cancer in which BALB/c mice received a single neonatal administration of BPA and at sexual maturity were injected with syngeneic 4T1 mammary adenocarcinoma cells, BPA exposure resulted in the formation of larger tumors infiltrated by a higher amount of Foxp3^+^ cells [94]. 

An additional key mechanism of tumor immune escape is based on the interaction between the immune inhibitory receptor PD-1, expressed on the membrane of T cells, and its ligand PD-L1, expressed on cancer cells and different cell types of the tumor microenvironment [57,58]. Activation of the PD-1/PD-L1 pathway impairs T cell activity, diminishes cytokines production, and prompts immune tolerance towards tumor cells, and the inhibition of this pathway appears as one of the most promising anticancer tools developed recently [57,95,96,97]. In this study, we show that although PD-L1 was expressed at similar levels in invasive tumors from CTR and BPA-treated mice, its cognate receptor PD-1 was instead significantly increased in the BPA-treated group. Moreover, we demonstrate that tumors from BPA-treated mice had a higher frequency of CD8^+^PD-1^+^ (i.e., exhausted) T lymphocytes. These findings highlight a further mechanism through which the endocrine disruptor can favor tumor escape from immune surveillance.

Collectively, these results extend and support the findings obtained in different tumor models, providing evidence that BPA exposure can affect breast cancer growth and progression by acting on tumor cells as well as on the tumor immune microenvironment [46,94,98,99], further suggesting that its effects should also be explored more generally in the context of cancer immunotherapy [47,100,101,102]. As regards the direct impact of BPA on tumor cells, our in vitro findings demonstrate that BPA has opposite, dose-dependent effects on the survival of TUBO cells, a cell line established from a BALB–*neu*T mouse breast carcinoma, with the lowest concentrations being the most effective in increasing tumor cell survival. These results, consistent with previous studies performed using different cancer cell lines [44,103,104,105], confirm that BPA’s impact on cancer progression can be particularly marked after chronic exposure to a low dose of the compound.

## 4. Materials and Methods

### 4.1. Reagents

Bisphenol-A (BPA, 2,2-Bis(4-hydroxyphenyl)propane, cat. no. 239658) and ethanol (EtOH) were purchased from Merck-Italy-Sigma Aldrich (St. Louis, MO, USA). For IHC analysis, antibodies against estrogen receptor alpha (αER) (cat. no. ab241557; 1:1000), progesterone receptor (PR) (SP42; cat. no. ab101688; 1:400), and anti-G-protein coupled receptor 30 (GPR30) (cat. no. ab260033; 1:200) were purchased from Abcam (Cambridge, UK). Antibodies against ErbB2/*neu* (C-18; cat. no. sc-284; 1:50), Akt (B-1; cat. no. sc- 5298; 1:100), phospho-Akt (S473) (C-11; sc-514032; 1:100), and Foxp3 (sc-53876; 1:100) were obtained from Santa Cruz Biotechnology (Santa Cruz, CA, USA). The anti-cleaved caspase 3 antibody (D175; cat. no. #9661S; 1:400) was purchased by Cell Signaling Technology (Danvers, MA, USA). Antibodies anti-PD-1 (cat. no. ab-84286; 1:75) and anti-PD-L1 (cat. no. ab-84131; 1:100) were purchased from Immunological Sciences (Rome, Italy). Antibodies anti-CD31/PECAM-1 (WM59; cat. no. #MA1-26196; 1:200), anti-CD8 (RIV11; cat. no. #MA1-7632; 1:100), and anti-CD4 (RIV6; cat. no. #MA1-7631; 1:100) were obtained from Thermo Fisher Scientific (Waltham, MA, USA). The anti-F4/80 (CI:A3-1; cat. no. #BE0206; 1:100) antibody was obtained from BioXcell (Lebanon, NH, USA) and the antibody against Ki67 (SP-6; cat. no. #MA5-14520; 1:250) from Invitrogen (Milan, Italy).

For Western blotting analysis, antibodies against Akt (C67E7; cat. no. #4691S; 1:1000) and phospho-Akt (S473) (D9E; cat. no. #4060S; 1:500) were obtained from Cell Signaling Technology (Danvers, MA, USA). Goat anti-rabbit IgG peroxidase-conjugated secondary antibody (cat. no. A6154; 1:10,000) was obtained from Merck-Italy-Sigma Aldrich (St. Louis, MO, USA).

### 4.2. Transgenic BALB–neuT Mouse Colony

Transgenic BALB–*neu*T male mice were routinely mated with BALB/c females (H-2d; Charles River, Calco, Italy) in the animal facilities of the University of Rome Tor Vergata. The progeny were confirmed for the presence of the transgene by PCR [106]. Mice were bred under pathogen-free conditions and handled in compliance with European Union and institutional standards for animal research under protocols approved by the Italian Ministry of Health (authorization no. 577-2022-PR).

### 4.3. Treatment of BALB–neuT Mice

Two groups of twelve individually tagged virgin females were used. Since their weaning (3 weeks of age) and up to sacrifice (30 weeks of age on average), mice were supplied with BPA (25 µg/L) or with its vehicle (EtOH 0.08%) in drinking water ad libitum. Water with BPA or EtOH vehicle alone was changed weekly, and the volumes drunk by mice in each cage were recorded. On a weekly basis, mice were weighed and mammary glands were inspected. Tumors were recorded at 3 mm in diameter, and tumor growth was monitored until all 10 mammary glands displayed a palpable mass or a single tumor mass exceeding 10 mm in diameter. At this point, or at earlier signs of distress, mice were sacrificed, and tumors and organs collected for analysis. The time of initial tumor appearance as well as tumor multiplicity, was averaged as the mean ± standard deviation of incidental tumors [50]. For tissue analysis at the preinvasive stage, four more groups of mice (n = 3 in each group) were similarly treated with BPA or vehicle up to 11 weeks of age, when mammary tissues were collected.

### 4.4. Histological Analysis and Immunohistochemistry

At sacrifice, mammary tumors from three animals from each group were used for histological examination after hematoxylin/eosin staining using 3 μm thick paraffin sections. IHC was used to assess the presence of αER, PR, cleaved caspase 3 or Ki67 positive cells and the expression of ErbB2/*neu*, GPR30, CD4, CD8, F4/80, CD31, Akt, and phospho-Akt in samples from control- and BPA-treated mice. Serial sections were sliced in order to have a more complete and similar histological frame. 

Briefly, antigen retrieval was performed on 3 μm paraffin sections of each sample using citrate pH 6.0 or EDTA citrate pH 7.8, for 30 min at 95 °C. Afterwards, sections were incubated for 1 h at room temperature with primary antibodies. The antibody–antigen binding was revealed by the Horseradish Peroxidase-3,3-diaminobenzidine (HRP-DAB) Detection Kit (cat. no. AFN600 and ACH500, UCS Diagnostic, Rome, Italy) [107,108]. PBS/Tween 20 pH 7.6 was used to remove non-specific bindings. The count of αER- and PR-positive cells was performed on tumor sections (20× objective) by two investigators in a blind fashion. Ki67, cleaved caspase 3, CD4, CD8, and F4/80 expression was estimated by counting the number of positive cells on 5 high power fields (HPF) (20×) by two investigators in a blind fashion. CD31 expression was estimated by counting the number of positive vessels on 5 high power fields (HPF) (20×). The number of positive cells per field was normalized by the total number of cells per field in order to ensure consistency across samples. ErbB2/*neu*, GPR30, Akt, and phospho-Akt expression were semi-quantitatively evaluated through a combined scoring system. Specifically, for each HPF, the total score (0–3) was obtained by adding the score associated with the number of positive cells to the score related to signal intensity. The score associated with the number of positive cells was defined as follows: 0 (0–1 positive cells/HPF), 1 (2–10 positive cells/HPF), 2 (11–20 positive cells/HPF), or 3 (≥21 positive cells/HPF). The score related to signal intensity was defined as follows: 0 (absent/very low intensity), 1 (low intensity), 2 (moderate intensity), or 3 (high intensity). Sections were observed and photographed using an Olympus BX53 microscope (Hachioji, Tokio, Japan).

### 4.5. Cell Extraction from Murine Tissues and Flow Cytometry Assay

Tumors and spleens were collected and processed to obtain a single-cell suspension, as previously described [107]. To extract tumor-infiltrating leukocytes, tumors were mechanically dissociated in PBS 2% FBS onto a 70-μm cell strainer (cat. no. 352350, Falcon, Thermo Fisher Scientific, Waltham, MA, USA) in a Petri dish, and leukocytes were enriched through 40/80 Percoll (cat. no. 17089101, GE Healthcare, Chicago, IL, USA) density gradient. Splenocytes were obtained by mechanical dissociation, followed by incubation with Red Blood Cell Lysis Buffer (cat. no. 11814389001, Roche, Basel, Switzerland) for erythrocyte lysis. Cells (5 × 10^5^) were stained with Fixable Viability Dye eFluor780 (cat. no. 65086514, eBioscience, Thermo Fisher Scientific, Waltham, MA, USA), and the following antibodies for surface markers from Sony Biotechnology Inc. were used: CD4 FITC (clone RM4-5, cat. no. #1102550), CD3 FITC (clone 17A2, cat. no. #1101020), CD8a PE (clone 53–6.7, cat. no. #1103540), CD25 PE-Cy7 (clone PC61, cat. no. #1110080), PD1 AF-647 (clone 29F.1A12, cat. no. #1276150), F4/80 PE (clone BM8, cat. no. #1215550), and Cd11b BV-510 (clone M1/70, cat. no. #1106225). Cells were fixed/permeabilized with Foxp3/Transcription Factor Staining Buffer Set according to the manufacturer’s instructions (cat. no. 00-5523-00, eBioscience), and staining was performed with Foxp3 AF-700 (clone MF-14, cat. no. #1232110, Sony Biotechnology Inc., San Jose, CA, USA). Acquisition of 50,000 cells/sample in the lymphocytes’ gate was performed on a CytoFLEX flow cytometer (Beckman Coulter, Brea, CA, USA). Samples were analyzed using CytExpert version 2.5 software (Beckman Coulter).

### 4.6. Cell Lines and Treatment

BALB–*neu*T mammary cancer cells (H-2^d^) (TUBO) that overexpress activated rat ErbB2/*neu* were kindly provided by Prof. G. Forni and Prof. F. Cavallo (University of Turin, Torino, Italy) and were maintained in Dulbecco’s modified Eagle medium (DMEM) high glucose without phenol red containing 20% fetal bovine serum, 100 U/mL penicillin and 100 μg/mL streptomycin (complete medium) (all purchased from Aurogene, Rome, Italy) [49,109]. Cells were cultured at 37 °C in a humidified incubator with 5% CO_2_. BPA was dissolved in 0.5% EtOH. For treatments, cells were incubated for the indicated times in the presence of BPA at different concentrations (dose range: 0.1–100 µM) or 0.5% EtOH as control (CTR).

### 4.7. Sulforhodamine B Assay

The sulforhodamine B (SRB) assay, performed as previously described [110], was used to assess the survival of TUBO cells exposed to BPA. Briefly, TUBO cells were seeded in flat-bottomed 96-well plates at 2500 cells/well. After 24 h, cells were treated for 24, 48, and 72 h with increasing concentrations of BPA (0.1, 1, 10, and 100 µM) or 0.5% EtOH as the control in a complete culture medium. Cells were then fixed with cold trichloroacetic acid (TCA, final concentration 10%, cat. no. T0699, Merck-Italy-Sigma Aldrich) for 1 h at 4 °C, washed in distilled water, air dried, and stained for 30 min using 0.4% (*w*/*v*) SRB (cat. no. S1402, Merck-Italy-Sigma Aldrich) solution in 1% acetic acid. After four washes with 1% acetic acid, the plate was allowed to dry. Finally, the dye was dissolved by adding 100 µL of 10 mM Tris pH 10 per well. The optical density (O.D.) of the samples was determined at 540 nm with a spectrophotometric plate reader. The percentage survival of BPA-treated cultures was determined by normalizing their O.D. values to those of the control cultures treated with EtOH [111]. The experiments were performed in triplicate and repeated three times.

### 4.8. Western Blotting

1 × 10^6^ TUBO cells were seeded in 100-mm tissue culture dishes 24 h prior to the addition of BPA (0.1–1 µM) or EtOH 0.5% (CTR). After 24 h of treatment, cells were lysed, and 80 µg of cell lysates were resolved in 12% SDS-PAGE and then transferred to nitrocellulose membranes. After blocking, the membranes were incubated with specific primary antibodies at 1–2 µg/mL concentrations overnight at 4 °C. After being washed, the filters were incubated with goat anti-rabbit IgG peroxidase-conjugated antibody and developed by enhanced chemiluminescence system ECL LiteAblot (cat. no. EMP011005, Euroclone, Milan, Italy) as previously described [97]. Densitometric analysis of autoradiographic bands was performed with Image J software 1.53e (National Institutes of Health, Bethesda, MD, USA) after blot scanning and expressed as bar graphs in the figures.

### 4.9. Statistical Analysis

Differences in mice weight, tumor weight, and multiplicity were evaluated by a two-tailed Student’s *t*-test. Survival curves were analyzed using the Kaplan–Meier method and compared with a log-rank test (Mantel-Cox) [112]. IHC scores and frequency of flow cytometric data were compared by a two-tailed Student’s *t-*test. Data distribution of cell survival assays was preliminarily verified using the Kolmogorov–Smirnov test, and the data sets were analyzed using one-way analysis of variance (ANOVA) followed by the Newman Keuls test. Differences in the intensity of immunoreactive bands were analyzed by a two-tailed Student’s *t*-test. Statistical analyses were performed using the GraphPad Prism software (version 6.0, La Jolla, CA, USA) with the significance threshold set at *p* ≤ 0.05.

## Data Availability

The original contributions presented in the study are included in the article and supplementary material, further inquiries can be directed to the corresponding author/s.

## Supplementary Materials

The following supporting information can be downloaded at https://www.mdpi.com/article/10.3390/ijms25116259/s1.
