# Supplementary material for: Bisphenol-A in Drinking Water Accelerates Mammary Cancerogenesis and Favors an Immunosuppressive Tumor Microenvironment in BALB–neuT Mice
Source: Int J Mol Sci. 2024 Jun 6;25(11):6259. doi: 10.3390/ijms25116259 (PMC11172679; doi:10.3390/ijms25116259)

Supplementary figure 1. Histological characterization of mammary tumor tissues from BALB-neuT mice. Mammary tissues were collected from BPA-treated and untreated (CTR) BALB-neuT mice at the preinvasive or invasive stage of tumor progression (n = 3). Hematoxylin/eosin (H/E) staining. Images were acquired with an OLYMPUS BX53 microscope (original magnification 100×).

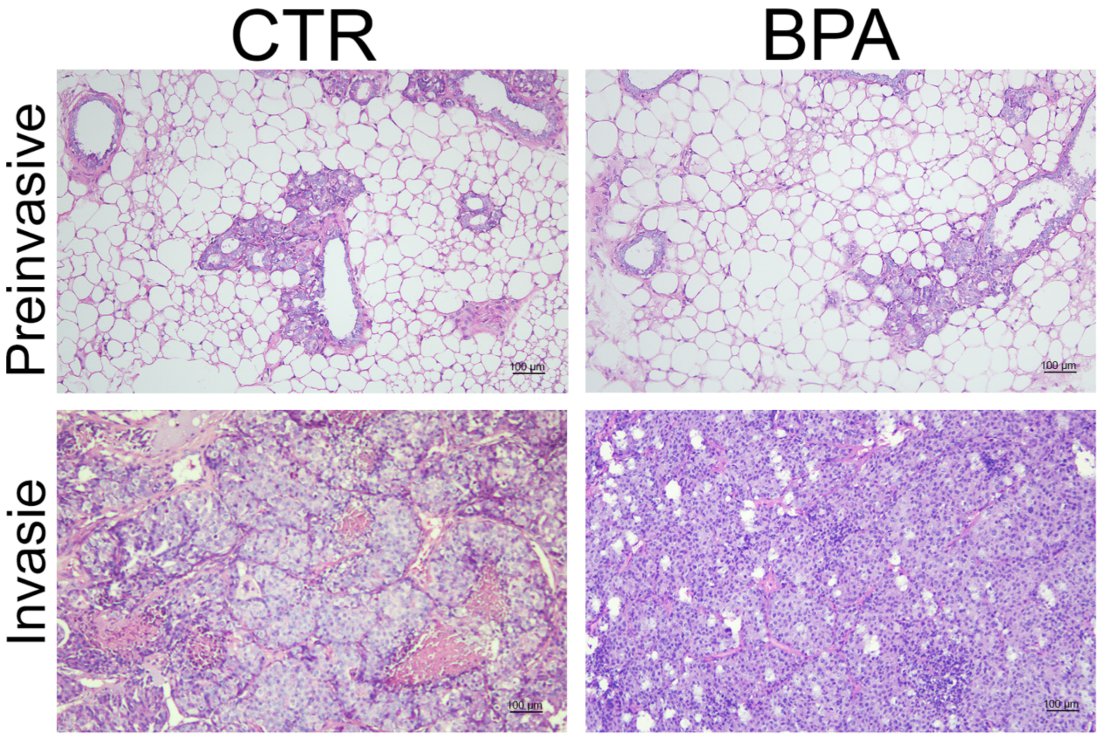

Supplement: Supplementary file 1 [file ijms-25-06259-s001.zip › ijms-2986136-supplementary.pdf]
